# A population-based estimation of maternal mortality in Lagos State, Nigeria using the indirect sisterhood method

**DOI:** 10.1186/s12884-024-06516-w

**Published:** 2024-04-25

**Authors:** Kikelomo Ololade Wright, Temiloluwa Fagbemi, Victoria Omoera, Taiwo Johnson, Adedayo Ayodele Aderibigbe, Basit Baruwa, Folashade Oludara, Olusegun Ogboye, Donald Imosemi, Olufemi Omololu, Babatunde Odugbemi, Oluwatoni Adeyemi, Adenike Omosun, Ibironke Akinola, Modupe Akinyinka, Mobolanle Balogun, John Abe, Bamidele Sadiku, Aduragbemi Banke-Thomas, Adetokunbo O. Fabamwo

**Affiliations:** 1 Department of Community Health and Primary Health Care, Lagos State University College of Medicine (LASUCOM), Ikeja, Lagos Nigeria; 2 Centre for Reproductive Health Research and Innovation (CHRHI), LASUCOM, Ikeja, Lagos Nigeria; 3 Department of Community Health and Primary Health Care, Lagos State University Teaching Hospital (LASUTH), Lagos, Nigeria; 4 Directorate of Family Health and Nutrition, Lagos State Ministry of Health (LSMoH), Lagos, Nigeria; 5 Lagos Bureau of Statistics (LBS), Lagos, Nigeria; 6 Onikan Health Centre, Lagos, Nigeria; 7 Island Maternity Hospital, Lagos, Nigeria; 8 Department of Planning, Research and Statistics, Lagos State Health Service Commission, Lagos, Nigeria; 9 Department of Paediatrics and Child Health, LASUCOM, Lagos, Nigeria; 10 Department of Community Health and Primary Care, College of Medicine, University of Lagos, Lagos, Nigeria; 11 Department of Demography and Social Statistics, Obafemi Awolowo University, Ile-Ife, Osun Nigeria; 12 National Population Commission, Abuja, Nigeria; 13 Faculty of Epidemiology and Population Health, London School of Hygiene and Tropical Medicine, London, UK; 14 Department of Obstetrics and Gynaecology, LASUTH, Lagos, Nigeria

**Keywords:** Maternal mortality ratio, Sisters, Maternal causes

## Abstract

**Background:**

Pregnancy and delivery deaths represent a risk to women, particularly those living in low- and middle-income countries (LMICs). This population-based survey was conducted to provide estimates of the maternal mortality ratio (MMR) in Lagos Nigeria.

**Methods:**

A community-based, cross-sectional study was conducted in mapped Wards and Enumeration Areas (EA) of all Local Government Areas (LGAs) in Lagos, among 9,986 women of reproductive age (15–49 years) from April to August 2022 using a 2-stage cluster sampling technique. A semi-structured, pre-tested questionnaire adapted from nationally representative surveys was administered using REDCap by trained field assistants for data collection on socio-demographics, reproductive health, fertility, and maternal mortality. Data were analysed using SPSS and MMR was estimated using the indirect sisterhood method. Ethical approval was obtained from the Lagos State University Teaching Hospital Health Research and Ethics Committee.

**Results:**

Most of the respondents (28.7%) were aged 25–29 years. Out of 546 deceased sisters reported, 120 (22%) died from maternal causes. Sisters of the deceased aged 20–24 reported almost half of the deaths (46.7%) as due to maternal causes, while those aged 45–49 reported the highest number of deceased sisters who died from other causes (90.2%). The total fertility rate (TFR) was calculated as 3.807, the Lifetime Risk (LTR) of maternal death was 0.0196 or 1-in-51, and the MMR was 430 per 100,000 [95% CI: 360–510].

**Conclusion:**

Our findings show that the maternal mortality rate for Lagos remains unacceptable and has not changed significantly over time in actual terms. There is need to develop and intensify community-based intervention strategies, programs for private hospitals, monitor MMR trends, identify and contextually address barriers at all levels of maternal care.

## Introduction

One of the global health development targets as outlined in the Sustainable Development Goals (SDGs) is the reduction of the global maternal mortality ratio (MMR) to less than 70/100,000 by 2030 [1, 2]. Although global MMR has decreased by 34% worldwide between 2000 and 2020 [3], pregnancy and childbirth continue to pose a significant risk of mortality to women, particularly those living in low- and middle-income countries (LMICs) as they are often confronted with numerous obstacles [4]. The World Health Organization (WHO) and its partners reported that approximately 287,000 mothers died in 2020, with 99% of these deaths occurring in LMICs [5]. Of these deaths, Nigeria, which is the most populated country in sub-Saharan Africa, was responsible for 30% of global maternal-related deaths [6].

According to the 2018 Nigeria Demographic and Health Survey (NDHS), there is an estimated 512 maternal deaths per 100,000 live births [95% CI: 447–578] in Nigeria [7]. Lagos, one of the largest cities in Africa with over 20 million people, is located in Nigeria [8]. A survey conducted by the Campaign Against Unwanted Pregnancy (CAUP) in 2010 reported that Lagos State had an estimated MMR of 555/100,000 [9, 10]. Another community-based study carried out in Lagos in 2011 estimated MMR to be 450/100,000 [95% CI: 360–530] [11]. However, higher estimates have been reported within the state. For instance, a 2012 study conducted in two urban slums reported an estimated MMR of 1,050/100,000 [95% CI: 894–1215] in 2017 [12].

Countries with the highest rates of maternal morbidity and mortality often report the least dependable data on maternal health [13]. Furthermore, facility-based studies of maternal mortality estimates are sometimes incomplete as they do not account for unreported maternal deaths or those occurring in the community. Also, costs associated with direct measurements of MMR as well as the absence of reliable vital statistics have necessitated the utilization of other methods including the sisterhood method, which has been useful for MMR estimation within and outside Nigeria [14–16]. Population and community-based surveys have proven to be effective in assessing maternal health services, providing valuable information for the design and evaluation of programs and strategies to improve women’s health [17]. As there are no sub-national estimates in the NDHS and the last estimate at the state level was published over 12 years ago, it was essential to estimate a more up-to-date MMR to track progress, guide policy decisions and planning for maternal health as the world strives towards attaining global targets. This survey was conducted as part of a larger study to assess population indices and services in the Lagos State region. Its primary objective is to provide time point estimates that can be used to evaluate maternal health programs and implementation strategies. Specifically, the survey aims to provide updated and representative estimates of maternal mortality in Lagos. The findings of this survey will be instrumental in identifying areas that require improvement and enhancing the effectiveness of maternal health programs.

## Materials and methods

### Study setting

Situated in south-west Nigeria, Lagos is a cosmopolitan city and the nucleus of the Nigerian economy. It is made up of five administrative divisions and twenty Local Government Areas (LGAs) which are further divided into wards and enumeration areas (EAs).

### Study design and sampling

This community-based, cross-sectional study was conducted in all the twenty LGAs of Lagos State amongst women of reproductive age (15–49 years) between April and August 2022 using the indirect sisterhood method which is an alternative and less expensive method for estimating maternal mortality in settings where direct data sources are inadequate or unreliable. This method utilizes information on the proportion of deaths of adult sisters during pregnancy, delivery or postpartum reported by adults in the course of a survey [18].

### Sample size estimation

Using the indirect sisterhood method, in settings with high levels of maternal mortality (over 500 maternal deaths per 100,000 live births), minimum sample sizes required to provide reliable estimates can be of the order of 4,000 households or less for adult respondents with an error margin of 20.0% [19]. In using a cluster sampling technique, a design effect of 2.5 was used as the correction factor to adjust the sample size as follows:

$$4000\text{*}2.5\hspace{0.17em}=\hspace{0.17em}\text{10,000}$$

A sample size of 10,000 consenting households comprising women within the reproductive age groups of 15–49 years was selected across all LGAs of the state for the survey. This was distributed as 500 women in the households per LGA (* 20 LGA) using a two-stage sampling process as follows:

Stage 1: The LGAs consist of Wards and EAs as demarcated by the National Population Commission (NPC). Ten wards were selected from each LGA using a simple random sampling technique. Subsequently, clusters of EAs in each ward were selected with the Probability Proportion to Size (PPS) of a geographic area.

Stage 2: In each cluster, household selection was conducted by visiting all households to determine the presence of eligible women. This process continued until all enumeration areas were covered. To attain a representative sample from each LGA, a total of 500 households from 10 EAs were mapped per LGA, ensuring geographic spread. Thereafter, consenting respondents were selected consecutively from eligible households for face-to-face interviews.

### Data collection

To collect data on maternal mortality, a questionnaire adapted from two commonly used surveys (Demographic Health Survey [DHS] and Multiple Indicator Cluster Survey [MICS]) [19, 20] was administered by trained field assistants. The survey tool was used to collect socio-demographic information on respondents and their deceased sisters. The indirect sisterhood method was utilized to gather data on maternal deaths and related factors [18]. Some questions asked include:

1. How many children did your mother give birth to?
2. How many sisters have you ever had, born to the same mother who were ever married (or who attained the age 15)?
3. How many of these sisters are dead?
4. How many of these sisters died during pregnancy, childbirth or two months after delivery?

The proportion of sisters within the reproductive age group for this study was estimated from the Lagos State age-sex 2022 projection data curated by the Lagos Bureau of Statistics (LBS). The study tool was pre-tested to refine the instrument and subsequently interviewer-administered to participants to ensure that the questions were properly understood by respondents.

### Data management

Real-time data collection and management were facilitated through the utilization of the Research Electronic Data Capture (REDCap) [21, 22]. To enhance the accuracy of the collected data, personal identifying information for the data collectors - including their names, initials, signatures, and GPS coordinates - was programmed on the tablets used for data collection for daily uploads.

### Data analysis

Data were analysed using SPSS (IBM SPSS Statistics for Windows, Version 26.0. NY).

The total fertility rate (TFR) was calculated by summing up the age-specific fertility rate for women in each age group, multiplying the total by 5 and then dividing by 1,000.

To obtain the TFR for the last 3 years, the initially calculated TFR was multiplied by 0.6.

Sister units for risk of exposure (Bi) were calculated by multiplying the number of ever-married sisters (Ni) by an adjustment factor for each age group. The adjustment factors used for the calculation of Bi are 0.107, 0.206, 0.343, 0.503, 0.664, 0.802, and 0.900 for the age range 15–19 years, 20–24 years, 25–29 years, 30–34 years, 35–39 years, 40–44 years and 45–49 years respectively [11, 18, 23].

The lifetime risk of maternal death (LTR or Q) was calculated by dividing the number of sisters’ deaths from maternal causes by sister units for risk of exposure [23].

To calculate 95% CI for Q, the following formula was utilized:

95% CI for Q = Q ± 1.96√(Q)*(1-Q)/B [24].

MMR was calculated using the formula: MMR = 1 – [(1 – Q)^1/1.2*TFR^] [25].

Sensitive questions were asked discreetly, and participants were assured of confidentiality. Those who appeared traumatized by recounting the loss of their loved ones were counseled before continuing with the survey. Also, respondents were informed of their right to withdraw from the study without any consequence.

## Results

Results from 9,986 women between the ages of 15 and 49 years (Table 1) are presented, excluding women with missing ages and those outside the age group under consideration.

**Table 1:** Socio-demographic characteristics of respondents

Age group	n (%)
15–19	340 (3.4)
20–24	1153 (11.5)
25–29	2862 (28.7)
30–34	2751 (27.5)
35–39	2035 (20.4)
40–44	664 (6.6)
45–49	181 (1.8)
Total	9986

Most of the respondents aged 25–29 years (28.7%) reported the highest proportion (32.1%) of.

ever-married sisters (Table 2). Of 546 deceased sisters reported, 120 (22.0%) died from maternal causes. Respondents aged 20–24 had the highest number of sisters who died from maternal causes (46.7%), while those aged 45–49 reported the highest number of sisters who died from other causes (90.2%).

The study findings showed that over the past three years, the total fertility rate (TFR) was 3.807. Additionally, there were 430 maternal deaths per 100,000 live births, with a 95% confidence interval of 360 to 510. The lifetime risk of maternal mortality (LTR) was 0.0196, which translates to a one in fifty-one (1-in-51) chance of dying during pregnancy or childbirth.

**Table 2:** Computations of sister units’ risk of exposure, LTR and MMR

Age group	Number of respondents	Number of ever married sisters = Ni N (%)	Number of maternal deaths = ri	Number of deaths due to other causes	Total sisters dead	Proportion of maternal deaths (%)	Adjustment factor = Ai	Sister units of risk of exposure = Bi=(Ni*Ai)	Lifetime risk of dying a maternal death Q = ri/Bi=
15–19	340	2,614 (26.2)	1	3	4	25.0	0.107	279.698	0.0036
20–24	1153	3,178 (31.8)	14	16	30	46.7	0.206	654.668	0.0214
25–29	2862	3,209 (32.1)	22	85	107	20.6	0.343	1100.687	0.0200
30–34	2751	2,289 (22.9)	26	117	143	18.2	0.503	1151.367	0.0226
35–39	2035	1,716 (17.2)	37	111	148	25.0	0.664	1139.424	0.0325
40–44	664	1,243 (12.4)	16	57	73	21.9	0.802	996.886	0.0160
45–49	181	889 (8.9)	4	37	41	9.8	0.900	800.1	0.0050
	9986	15,138	120	426	546	22.0		6122.83	0.0196

## Discussion

Maternal mortality data can enable policymakers and healthcare professionals to identify areas that require improvement, leading to the development of targeted interventions and ultimately better health outcomes for mothers and their babies. In this study, we used the indirect sisterhood method to estimate MMR in Lagos. Most of the participants (over 80%) were within the 20–39 age group. Among the sisters of the participants, 22.0% had died from maternal causes. The lifetime risk of maternal mortality was 0.0196 [95% Confidence Interval (CI): 0.01613–0.02307], and the MMR was 430/100,000 [95% CI: 360–510].

The proportion (22.0%) of maternal deaths in our study is slightly lower than a similar study conducted in Lagos State in 2011 (23.4%) [11]. Further, our study found that sisters of the deceased aged 20–24 reported that almost half of the deaths (46.7%) were due to maternal causes. This finding is consistent with the results of studies conducted in two other states of the country that used the sisterhood method, which reported 46.8% and 36.4% of deaths due to maternal causes [23, 26].

Our findings showed that the likelihood of dying from a maternal cause is 1 in 51, with estimates ranging from 1 in 62 to 1 in 43. This figure may indicate a slight improvement in comparison to the 1 in 18 reported by a 2017 study in Lagos and the 1 in 22 found by WHO [12, 27]. However, it is still extremely high compared to rates in other developing (1 in 180) and developed (1 in 4900) countries [27, 28], highlighting the urgent need for interventions to close the gap and achieve global targets.

An MMR of 430 per 100,000 [95% CI: 360–510] found in this study indicates a minimal absolute reduction from the state-wide estimate of 450 per 100,000 [95% CI: 360–530] reported more than a decade ago [11]. However, our estimates are within the confidence interval of the previous study, suggesting that there is no statistically significant difference in MMR in the state and any difference observed is entirely due to chance. Crucially, the State Government has implemented a multi-pronged approach to reduce MMR over the years [10, 29], however, these do not appear to have made any significant difference. Generally, contributory factors to the lack of significant reduction in MMR may range from socio-contextual influences such as cultural beliefs, and care-seeking behaviour of women during the antenatal, intrapartum, and postnatal periods [30] to health system factors such as maternal health services being unavailable, insufficient, or underutilised [31, 32]. As our study was conducted at a time of a pandemic, which has been shown to have contributed to excess maternal deaths [33, 34], this might explain the lack of statistically significant difference over the past decade.

There are gaps between the efforts to improve MMR and the progress made so far in Lagos. Despite the establishment of more Mother and Child Centres (MCC) within the state and the improvement in capacity building of health service providers in government-owned facilities, there has not been significant improvement in MMR. This indicates the need to consider the private sector’s role in providing healthcare services and other contextual factors, including community-related factors outside the existing facility-based interventions. This is important because many maternal deaths possibly occur within the communities.

### Implications

Given the gaps between maternal health efforts and desired pregnancy outcomes identified from our findings, there is a need to critically assess and evaluate the implementation of existing maternal health initiatives and integrate alternative approaches to enhance the outcomes of these interventions and ultimately, improve maternal health more efficiently and effectively. Optimising maternal health care, integrating and regulating private hospitals and traditional birth attendants (TBAs), introducing community-based programs and behavioural change communication (BCC) programs as well as thorough monitoring of all maternal health services and pregnancy mortality surveillance are essential to ensuring every woman of childbearing age has access to quality care, when and where needed. Intervention strategies including improvement in regulatory mechanisms targeting the private sector are invaluable in addressing some of these challenges considering that the majority of the women had their last deliveries in private hospitals.

The need to routinely estimate maternal mortality in tracking progress made in MMR reduction cannot be overemphasized. Further research projects may consider estimating MMR at the LGA level, which will necessitate the utilization of more direct methods to obtain near-precise sub-state estimates. Furthermore, the use of varying methods of estimating MMR may complement the sisterhood method providing additional contextual insights.

The strength of this study is in the utilization of a methodological approach to engage women across all the LGAs of Lagos State providing an overall state estimate. However, sub-state estimates could not be ascertained on account of the limited sample size per LGA. Also, since we relied on verbal reports, the validity of our findings is limited by the extent of the truthfulness of our respondents. The authors also acknowledge that another limitation of this study is the non-identification of specific causes of maternal deaths. To the best of our knowledge, no other state has conducted a similar survey in Nigeria at a sub-national level, relying mostly on nationally representative surveys such as the Demographic Health Surveys (DHS) with limited samples from each state.

## Conclusion

Our findings show that the MMR for Lagos has not changed in actual terms and the numbers continue to be unacceptable. It is important to develop and intensify community-based intervention strategies, programs for private hospitals, monitor MMR trends, identify and contextually address barriers while reflecting on what can be done differently to see outputs that are commensurate with efforts invested in improving maternal health.

The funders had no role in study design, data collection and analysis, decision to publish, or preparation of the manuscript.

## Data Availability

Availability of data and materials - The datasets generated and/or analyzed during the current study are not publicly available and are the property of the Lagos State but are available from the corresponding author on reasonable request.Data supporting this study are available, subject to necessary approvals from the funder.
